# Supplementary material for: The First Heterozygous TWNK Nonsense Mutation Associated with Progressive External Ophthalmoplegia: Evidence for a New Piece in the Puzzle of Mitochondrial Diseases
Source: Biomolecules. 2025 Sep 18;15(9):1337. doi: 10.3390/biom15091337 (PMC12467334; doi:10.3390/biom15091337)
Supplement: Supplementary file 1 [file biomolecules-15-01337-s001.zip › biomolecules-3843784-supplementary.pdf]

Supplementary Materials

Original Western blot images relative to Figure 3b,c.

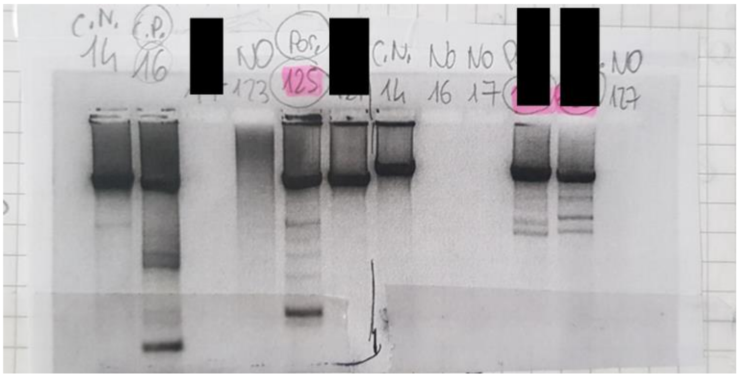

Figure S1. Original Western blot images relative to Figure 3b.

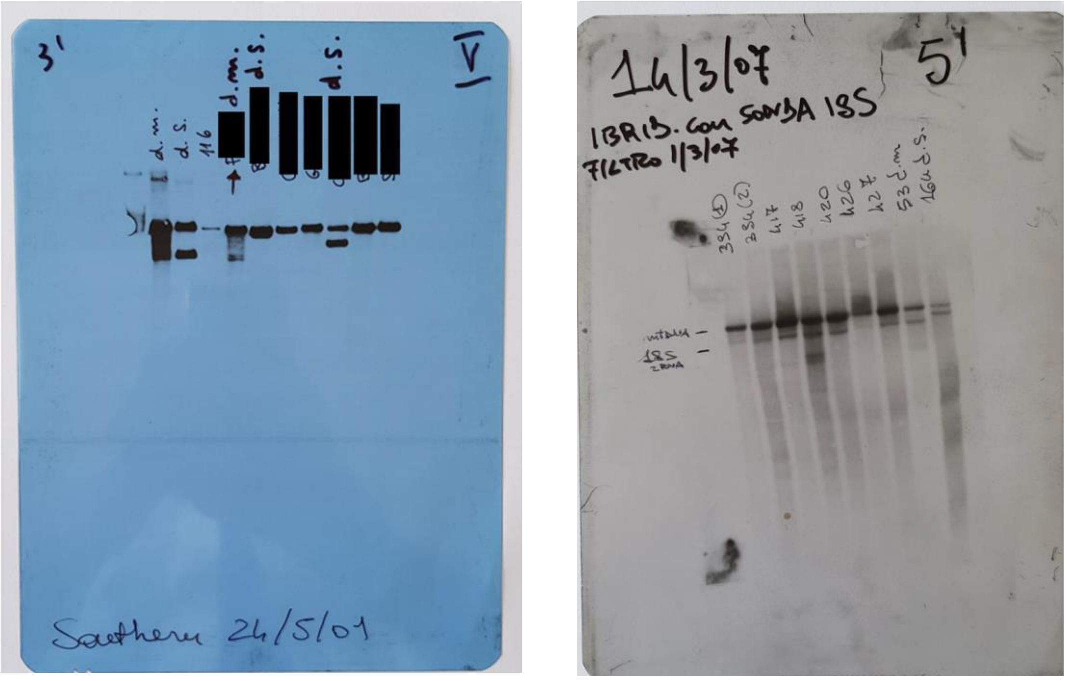

Figure S2. Original Western blot images relative to Figure 3c.
